# First experiences in the treatment of juveniles and idiopathic scoliosis with SpineCor braces

**DOI:** 10.1186/1748-7161-8-S1-P11

**Published:** 2013-06-03

**Authors:** A Sarchioto

**Affiliations:** 1 ASL BN1, Benevento, Italy

## Background

The standardized treatment of juvenile, and adolescent, idiopathic scoliosis is accepted everywhere: physiotherapy for curves until 15-20° Cobb, rigid brace between 20 and 30-35° Cobb, cast between 30 and 40-45° Cobb and surgery over 45° Cobb.

## Aim

The aim of this work is to verify the efficacy and effectiveness of the SpineCor dynamic brace, in juvenile and adolescent idiopathic scoliosis with curves between 20 and 50° Cobb.

## Methods

All patients (range of age 5 to 15 years) were treated with a SpineCor dynamic brace. All braces were ordered, fitted and used, following the standard canons of the appropriate procedure. A photographic control was carried out, just after fitting, a clinical control in a month, a clinical and photographic in three months, and a clinical, photographic and radiographic in six months.

## Results

Over 90% of patients had a very important change in their posture and cosmetic appearance. None of them left the treatment.

## Conclusion

The SpineCor dynamic brace used is efficient and effective. Because it does not limit any movement, and allows practicing all sports, (but swimming), and dance, and since it is virtually invisible under clothing, no patient has complained of the treatment. Both patients and parents were satisfied. This good result allows, and encourages, us to continue in using this brace.
